# Telaprevir or boceprevir in HIV/HCV-1 co-infected patients in a real-life setting. Interim analysis (24 weeks). COINFECOVA-SEICV study

**DOI:** 10.7448/IAS.17.4.19634

**Published:** 2014-11-02

**Authors:** Carlos Minguez, Enrique Ortega, Juan Flores, Jorge Carmena, Mar Masiá, Marta Montero, Sergio Reus, Carlos Tornero, Maria Jose Galindo, Miguel Garcia-Deltoro, Concepción Amador, Jose María Cuadrado, Jorge Usó, Jose López-Aldeguer

**Affiliations:** 1 Internal Medicine/Infectious Diseases Unit, Hospital General Universitario de Castellón, Castellón, Spain; 2 Infectious Diseases, Consorcio Hospital General Universitario de Valencia, Valencia, Spain; 3 Internal Medicina, Hospital Arnau de Vilanova, Valencia, Spain; 4 Internal Medicine/Infectious Diseases Unit, Hospital Universitario Dr Peset, Valencia, Spain; 5 Infectious Diseases, Hospital General Universitario de Elche, Elche, Spain; 6 Infectious Diseases, Hospital Universitari i Politecnic La Fe, Valencia, Spain; 7 Infectious Diseases, Hospital General Universitario de Alicante, Alicante, Spain; 8 Internal Medicina, Hospital Francesc de Borja, Gandia, Spain; 9 Infectious Diseases, Hospital Clinico Universitario de Valencia, Valencia, Spain; 10 Infectious Diseases, Hospital Comarcal de la Marina Baixa, Villajoyosa, Spain; 11 Infectious Diseases, Hospital Universitario San Juan de Alicante, Alicante, Spain; 12 Internal Medicine, Hospital Universitari i Politecnic La Fe, Valencia, Spain

## Abstract

**Introduction:**

In general, HIV co-infected patients included in clinical trials evaluating the hepatitis C virus (HCV) therapy with telaprevir (TVR) or boceprevir (BOC) with advanced fibrosis, are scarce. We analyze data concerning the use of these drugs in a real-life clinical setting with patients affected by a more advanced degree of fibrosis in a Spanish cohort.

**Methods:**

We evaluated safety and efficacy in an interim analysis encompassing the first 24 weeks of triple therapy with peginterferon (alfa-2a or alfa-2b), ribavirin and TVR or BOC in an observational, multicentre study. HIV/HCV genotype 1 co-infected patients beginning therapy from January 2012 to July 2013 were included.

**Results:**

Enrolled patients were 155 (144 patients on TVR and 11 on BOC), average age was 47 years, 83% were male. With respect to HCV treatment, 44% were naïve, 13% relapsers, 17% partial responders, 21% null responders, and in seven patients, the previous response was unknown. All but three (98%) were under antiretroviral therapy (ART) (other than reverse transcriptase inhibitors, the backbone was raltegravir 43%, atazanavir 35%, and etravirine 28%). Median HCV-RNA at baseline was 6.1 log10, 54% were cirrhotic and 38% F3. At week 4, 93% of patients continued on therapy, 81% at w12, and 73% at w24. Virological failure was observed more frequently in: cirrhotic patients (19% [95% CI, 11–27]) vs F3 (12% [CI, 4–20]); patients with TT allele of the IL28B polymorphism (40% [CI, 18–61]) vs CT (21% [CI, 12–31]), or CC (2,2% [CI, −2–6]); previous null responders (37.5% [CI, 21–54]) vs partial responders (15.4% [CI, 1–29]), naïve (13% [CI, 5–21]) or relapsers (0% [CI, 0–0]); and in patients with a genotype subtype 1a (23.6% [CI, 57–76]) vs 1b (8.1% [CI, −1–17]). Overall, 17% had virological failure and in 8% treatment was discontinued due to adverse events. Severe adverse events occurred in 30 patients (19%). Haematologic disorders were the most common type including severe anaemia in 12 (7.7%) patients. Erythropoietin was employed in 41 patients (26.4%) and 11 (7.1%) received blood transfusions. Nineteen patients (12.2%) were treated with G-CSF, and 17 (11%) with thrombopoietin-receptor agonists. Five patients died (3.2%), three due to hepatic decompensation, one due to pneumonia and one due to pulmonary hypertension.

**Conclusions:**

In a real-life setting, therapy against HCV in co-infected patients with advanced liver fibrosis shows high virologic success at 24 weeks. However, frequent haematologic disorders are observed and a close monitoring and an intensive therapy are needed to optimize the results.

